# Acute reduction of erector spinae muscle cross-sectional area is associated with ICU-AW and worse prognosis in patients with mechanical ventilation in the ICU: A prospective observational study

**DOI:** 10.1097/MD.0000000000027806

**Published:** 2021-11-24

**Authors:** Gang Yuan, Jie Zhang, Zhifang Mou, Jiye Luo, Yongpeng Xie

**Affiliations:** a Department of Radiology, Lianyungang Clinical College of Nanjing Medical University, The First People's Hospital of Lianyungang, Lianyungang, China; b Department of Critical Care Medicine, Lianyungang Clinical College of Nanjing Medical University, The First People's Hospital of Lianyungang, Lianyungang, China.

**Keywords:** ESMcsa, ICU-AW, mechanical ventilation, MRC-score, prognosis

## Abstract

**Background:**

To investigate the values of erector spinae muscle cross-sectional area (ESMcsa) loss for diagnosing intensive care unit-acquired weakness (ICU-AW) and predicting the 60-day survival status in patients with mechanical ventilation.

**Methods:**

Patients who were admitted into the intensive care unit (ICU) and received invasive mechanical ventilation therapy from June 2018 to June 2020 were selected. And they were divided into an ICU-AW group and a non-ICU-AW group, which were compared based on the ESMcsa and The British Medical Research Council muscle strength score (MRC-score) on the 1st and 7th day of ICU admission. The receiver operating characteristic curve was employed to measure the values of the ESMcsa Loss and ESMcsa Loss Ratio on the 7th day in order to diagnose ICU-AW in patients with mechanical ventilation. The survival curves of the patients were plotted to analyze the ESMcsa Loss Ratio values for predicting the 60-day survival status.

**Results:**

A total of 104 patients were enrolled, they were divided into the ICU-AW group (n = 56) and the non-ICU-AW group (n = 48). The mechanical ventilation time, ICU stay time, and hospital stay time of the ICU-AW group were all significantly higher than those of the non-ICU-AW group. On the 1st day, no significant difference in the ESMcsa or MRC-score between the 2 groups of patients was observed. On the 7th day, the ESMcsa and MRC-score of the ICU-AW group were significantly lower than those of the non-ICU-AW group. The ESMcsa Loss and ESMcsa Loss Ratio were both significantly negatively correlated with the MRC-score. The ESMcsa Loss and ESMcsa Loss Ratio on the 7th day were both valuable for the prediction of ICU-AW in patients with mechanical ventilation (areas under the receiver operating characteristic curve = 0.904, 0.835, and 0.889, *P* < .001). The survival rate of the patients in the high- and low-ESMcsa Loss Ratio groups were 60.0% and 80.0% (*P* < .05).

**Conclusions:**

As suggested by the ESMcsa Loss Ratios of the patients with mechanical ventilation on the 7th day of ICU admission, it offers a desirable objective indicator for the diagnosis of ICU-AW, and provides certain values for predicting the 60-day survival status of patients with mechanical ventilation in the ICU.

## 1 Introduction

Intensive care unit-acquired weakness (ICU-AW) is a common complication in critically ill patients with clinical manifestations such as difficult weaning, general weakness, tetraplegia, hyporeflexia (or even areflexia), and muscle atrophy. Apart from prolonging the stay time of patients,^[1]^ ICU-AW also leads to long-term dysfunction, increased mortality, and seriously affected prognosis.^[2,3]^ Studies have shown that the incidence of ICU-AW can reach as high as 60% in patients with mechanical ventilation in the intensive care unit (ICU).^[4]^ Therefore, the early diagnosis and timely intervention of ICU-AW are of vital importance. However, currently there are no available objective evaluation and prediction indicators for the assessment and diagnosis of ICU-AW.^[5,6]^ Some recently published foreign studies have suggested that erector spinae muscle cross-sectional area (ESMcsa) can be employed to assess the skeletal muscle mass in patients.^[7,8]^ As an observational study, this study aims to determine the connection between acute ESMcsa reduction and acute muscle wasting in patients with mechanical ventilation in the ICU, assess the ESMcsa values for the diagnosis of ICU-AW and the prediction of the 60-day survival status, and propose clinical intervention measures on this basis.

## 2 Study object and methods

### 2.1 Study object

Clear-minded patients with acute respiratory failure, who were admitted into the ICU of our hospital and received invasive mechanical ventilation therapy from June 2018 to June 2020, were selected after screening.

#### 2.1.1 Inclusion criteria

Age ≥18; in need for mechanical ventilation therapy; obeying commands to cooperate in the muscle test; estimated to have a mechanical ventilation therapy time of above 5 days; ICU stay time ≥7 days.

#### 2.1.2 Exclusion criteria

Glasgow coma scale below 15 or presence of motor weakness or neurological deficit such as reduced conscious level leading to unable to cooperate during examination, craniocerebral trauma, spinal cord injury, acute stroke; acute peripheral neuromuscular diseases, medical history of central cognitive dysfunction or neuromuscular diseases.

### 2.2 Ethics review and clinical trial registration

This study conformed to the standards of medical ethics and was approved by the ethics committee of the First People's Hospital of Lianyungang City (Approval No.: LCYJ20171228001). All patients were enrolled with the consent of their families, and in each case, a written informed consent was obtained. All methods were performed in accordance with the relevant guidelines and regulations, and the Chinese Clinical Trial Registry (ChiCTR) No.: ChiCTR1900025382 (25/08/2019).

### 2.3 Study methods

#### 2.3.1 Chest computer tomography scan and ESMcsa measurement

**Figure 1:**
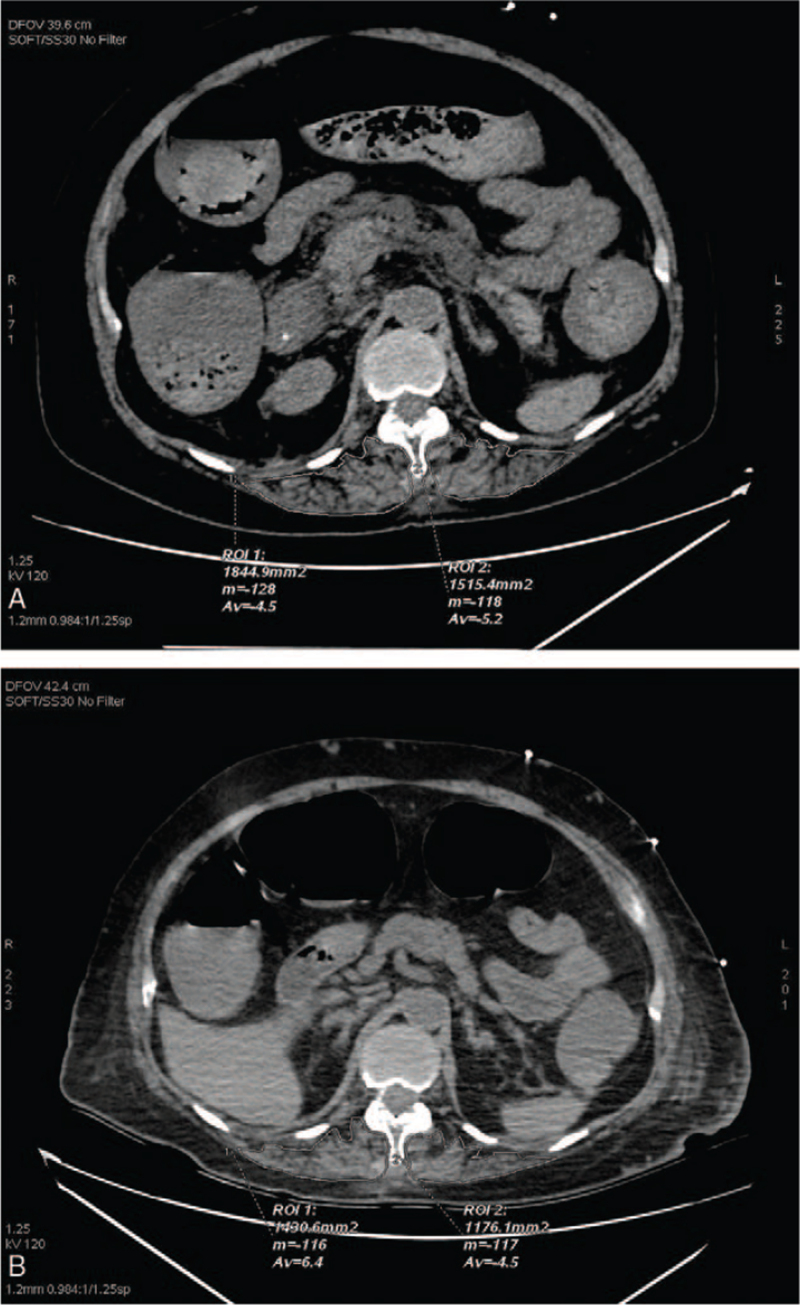
ESMcsa measurement and comparison in the same patient. ESMcsa = erector spinae muscle cross-sectional area.

All patients enrolled underwent chest computer tomography (CT) scan before admission or on the 1st day of admission, and chest CT re-examination after 7 days of treatment. The mediastinal window in the chest CT was used for the ESMcsa analysis. The inferior margin of the T-12 vertebra was targeted for the erector spinae muscle analysis.^[9]^ The ESMcsa measurement results were recorded in detail. The right and left erector spinae muscles were identified on the CT images, and pseudo-color images of the erector spinae muscles were delineated manually.^[10]^ The ESMcsa on each side and the total ESMcsa on both sides of the spine were calculated, as shown in Figure 1.

#### 2.3.2 Muscle strength assessment

All sedatives and analgesics were withdrawn on the 1st and 7th day of ICU admission. All patients should be conscious, so that they could cooperate during the examination. The British Medical Research Council muscle strength score (MRC-score) was adopted to assess 6 major muscle groups (bilateral wrist extension, forearm flexion, shoulder abduction, ankle joint dorsi-flexion, knee extension, and hip joint flexion) in the patients.^[11]^ The muscle strength of each muscle group was graded according to the Oxford muscle strength grading scale (aggregate score = 60). In clinical practice, an “aggregate score < 48” is adopted as the criterion for ICU-AW diagnosis.^[12]^ In this study, the muscle strength grading scores were given simultaneously by 2 trained physicians.

### 2.4 Grouping and statistical analysis

Data processing and mapping were performed using the Graphpad Prism 6.0 (San Diego, California) and SPSS 22.0 statistical software (IBM SPSS, Turkey). All measurement data were expressed in the form of (x ± s). Intergroup comparison was conducted by the 2 independent-samples *t* test. Enumeration data were compared using the χ^2^ test. *P* < .05 denoted statistical significance. The patients were divided into the ICU-AW and non-ICU-AW groups based on their MRC-scores on the 7th day of ICU admission. The ESMcsa and MRC-score of the 2 groups on the 1st and 7th day of ICU admission were compared. Pearson correlation analysis was performed on all patients to determine the correlation between ESMcsa Loss and MRC-score on the 7th day. The receiver operating characteristic (ROC) curve was employed to determine the effectiveness of the ESMcsa Loss and ESMcsa Loss Ratio on the 7th day in diagnosing ICU-AW in patients with mechanical ventilation. The Kaplan-Meier survival curves of the patients were plotted to analyze the ESMcsa Loss Ratio values for predicting their 60-day survival status.

## 3 Results

### 3.1 General clinical data comparison

Among the 166 patients selected after screening, 42 were excluded since they failed to meet inclusion criteria or met exclusion criteria. Among the 124 patients left, 14 were rejected due to that their stay time was <7 days or due to against-advice discharge or death, and 6 for not completing the chest CT re-examination and ESMcsa measurement on day 7. Ultimately, a total of 104 patients, including 65 males and 39 females, were enrolled. Baseline data were recorded, and the Sequential Organ Failure Assessment and 24 hours Acute Physiology and Chronic Health Evaluation scores were calculated. Statistical analysis concerned the mechanical ventilation therapy time, ICU stay time, and hospital stay time of the patients.

The ICU-AW and non-ICU-AW groups were compared in terms of baseline levels, such as gender, age, weight, body mass index, basic medical history, smoking history, cause of mechanical ventilation, Acute Physiology and Chronic Health Evaluation score, and Sequential Organ Failure Assessment score, and no statistically significant difference was observed (*P* > .05). Nevertheless, statistically significant differences between the 2 groups were observed in the mechanical ventilation therapy time, ICU stay time, and hospital stay time (*P* < .05), as shown in Table 1.

**Table 1:** Comparison of the baseline levels and clinical characteristics between the 2 groups of patients.

Indicator/group	ICU-AW group (n = 56)	Non-ICU-AW group (n = 48)	t/x^2^ value	*P* value
Age	62.05 ± 11.34	61.45 ± 12.51	0.257	.798
Female/Male	22/34	17/31	0.165	.685
Weight (kg)	64.42 ± 9.35	65.67 ± 11.45	0.613	.541
BMI (kg/m^2^)	24.45 ± 3.67	25.57 ± 3.66	1.553	.123
SOFA score	8.56 ± 3.28	8.35 ± 3.65	0.309	.758
APACHE II	18.25 ± 7.54	17.64 ± 8.01	0.400	.690
Smoking history	23/56	18/48	0.138	.710
Basic medical history	25/56	19/48	0.271	.603
Cause of MV				
Pulmonary infection	16/56	16/48	0.275	.599
AECOPD	20/56	14/48	0.504	.478
Pulmonary contusion	8/56	7/48	0.001	.966
ARDS	7/56	5/48	0.110	.740
Other	5/56	6/48	0.349	.555
Length of MV (h)	195.85 ± 52.32	161.34 ± 43.65	3.616	.001
ICU length of stay (days)	12.74 ± 3.35	11.04 ± 2.89	2.747	.007
Hospital length of stay (days)	18.98 ± 6.64	16.35 ± 4.86	2.295	.024

Basic medical history includes hypertension, diabetes, chronic kidney disease, surgery, etc. AECOPD = acute exacerbation of chronic obstructive pulmonary disease, APACHE II = Acute Physiology and Chronic Health Evaluation score, ARDS = acute respiratory distress syndrome, BMI = body mass index, ICU = intensive care unit, ICU-AW = intensive care unit-acquired weakness, MV = mechanical ventilation, SOFA = Sequential Organ Failure Assessment score.

### 3.2 Comparison of ESMcsa and MRC-score between the 2 groups of patients

**Figure 2:**
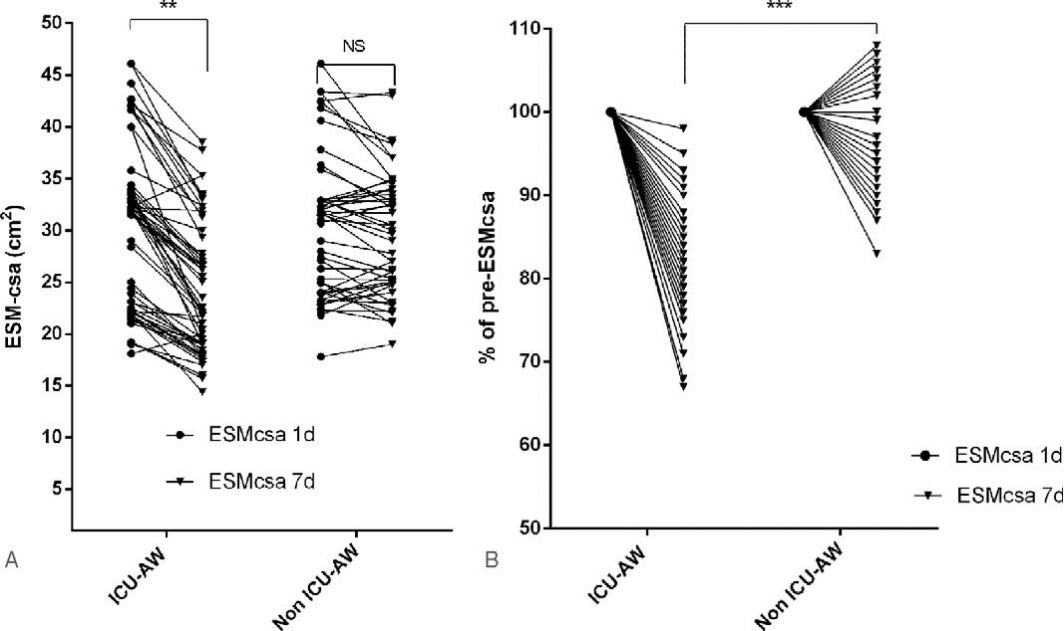
ESMcsa variation trends in the 2 groups of patients within 7 days. ESMcsa = erector spinae muscle cross-sectional area.

According to the intergroup comparison, no significant difference in the ESMcsa or MRC-score on the 1st day was found between the 2 groups of patients (*P* > .05). On the 7th day, the ESMcsa and MRC-score of the ICU-AW group were significantly lower than those of the non-ICU-AW group, and the differences were statistically significant (*P* < .05), as shown in Table 2. The ESMcsa of the ICU-AW group on the 7th day was significantly lower than that on the 1st day, indicating that the presence of acute muscle wasting reduced the skeletal muscle mass significantly. In contrast, in the non-ICU-AW group, muscle wasting was not significant, as shown in Figure 2.

**Table 2:** Comparison of the ESMcsa and MRC-score at different time points between the 2 groups of patients.

Indicator	Group	1st day	7th day
ESMcsa (cm^2^)	ICU-AW group (n = 56)	30.13 ± 7.54	23.68 ± 6.56
	Non-ICU-AW group (n = 48)	31.45 ± 6.89	27.43 ± 6.34
	t-value	0.926	2.951
	*P* value	.357	.004
MRC-score	ICU-AW group (n = 56)	54.34 ± 2.76	44.37 ± 3.59
	Non-ICU-AW group (n = 48)	53.56 ± 2.43	51.54 ± 2.24
	t-value	1.518	11.980
	*P* value	.132	.000

ESMcsa = erector spinae muscle cross-sectional area, ICU-AW = intensive care unit-acquired weakness, MRC-score = The British Medical Research Council muscle strength score.

### 3.3 Correlation analysis between ESMcsa Loss, ESMcsa Loss Ratio, and MRC-score

**Figure 3:**
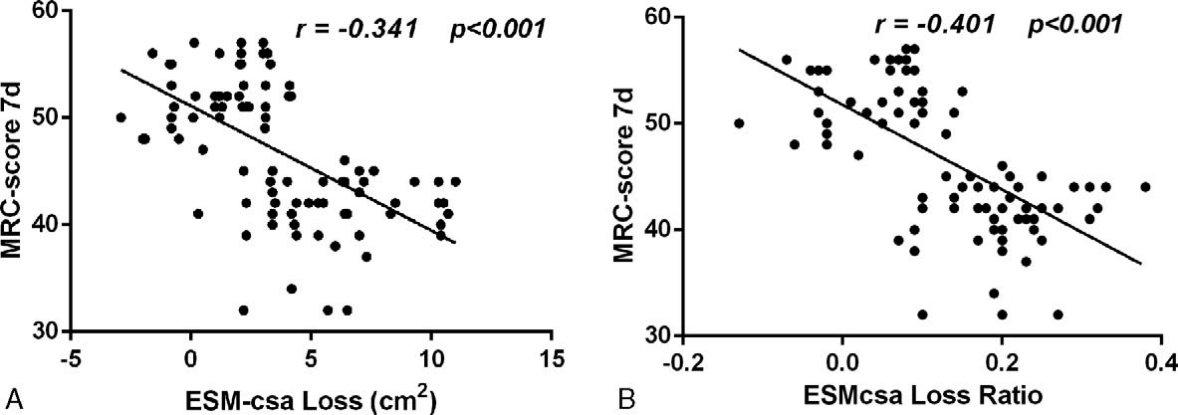
Correlation analysis between ESMcsa Loss, ESMcsa Loss Ratio, and MRC-score on the 7th day. ESMcsa = erector spinae muscle cross-sectional area, MRC-score = The British Medical Research Council muscle strength score.

The Pearson correlation analysis revealed that the ESMcsa Loss and ESMcsa Loss Ratio were both significantly negatively correlated with the MRC-score (R-values of –0.3072 and –0.3527; *P* < .001; Fig. 3A/B).

### 3.4 ESMcsa Loss and ESMcsa Loss Ratio values for the prediction of ICU-AW in patients with mechanical ventilation

**Figure 4:**
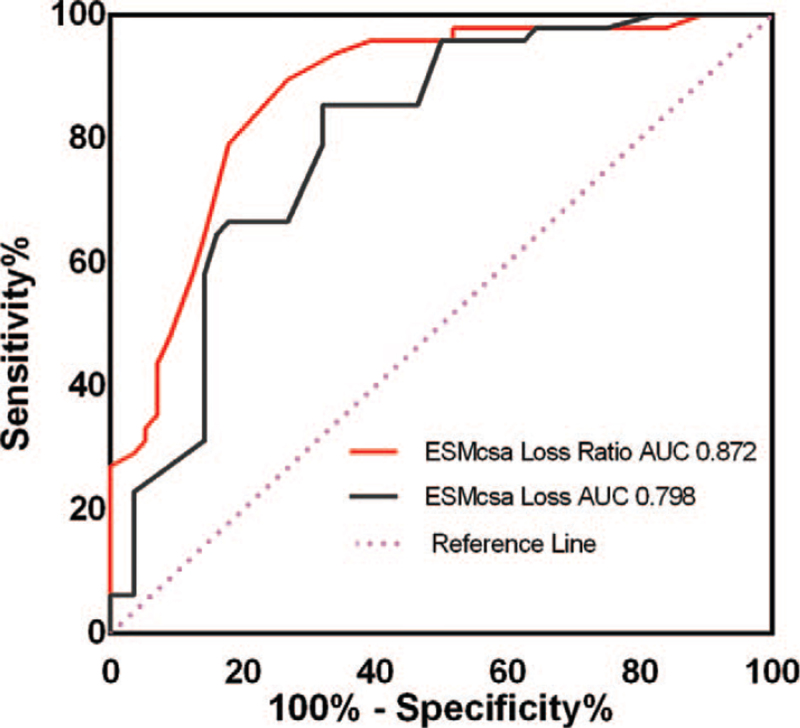
ROC curve in relation to ESMcsa Loss and ESMcsa Loss Ratio in ICU-AW diagnosis. ESMcsa = erector spinae muscle cross-sectional area, ICU-AW = intensive care unit-acquired weakness, ROC = receiver operating characteristic.

By analyzing the values of the ESMcsa Loss and ESMcsa Loss Ratio on the 7th day to diagnose ICU-AW in patients with mechanical ventilation using the ROC curve, it was found that, on the 7th day, when the areas under the curve (AUC) and optimal cut-off value of ESMcsa Loss were correspondingly 0.798 and 4.14 cm^2^, the sensitivity and specificity were 85.42% and 67.86%, respectively. When the AUC and optimal cut-off value of ESMcsa Loss Ratio were correspondingly 0.872% and 11.5%, the sensitivity and specificity were 89.58% and 73.21%, respectively (Fig. 4).

### 3.5 ESMcsa Loss Ratio values for the prognosis of patients with mechanical ventilation in the ICU

**Figure 5:**
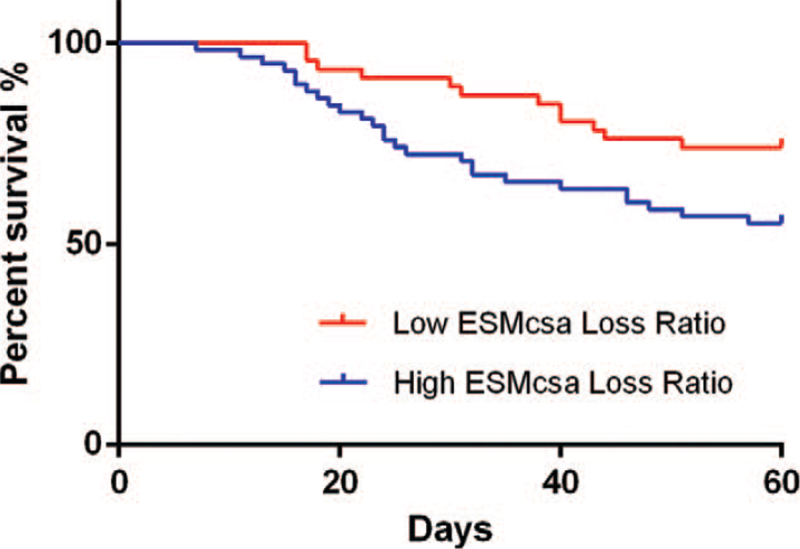
Survival rate curve in relation to ESMcsa Loss Ratio. ESMcsa = erector spinae muscle cross-sectional area.

Statistical analysis on the 60-day survival rate of all patients enrolled was performed. Based on the optimal cut-off value of ESMcsa Loss Ratio on the 7th day on the ROC curve, the patients were divided into a high-ESMcsa Loss Ratio group (n = 58) and a low-ESMcsa Loss Ratio group (n = 46). After plotting the survival curves of the patients, the 2 groups were compared in terms of the 60-day survival rate. The comparison results demonstrated that the survival rates of the patients in the high- and low-ESMcsa Loss Ratio groups were 55.17% and 73.91%, respectively, and the differences in the 60-day survival rate between the 2 groups were all statistically significant (*P* < .05), as shown in Figure 5.

## 4 Discussion

The incidence of ICU-AW in critically ill patients is high, but the state of illness is often concealed due to disturbance of consciousness on some of the patients or use of sedatives and analgesics,^[13]^ which further delay diagnosis and treatment. To this end, objective evaluation and selection of prediction indicators are very important.^[14]^

In this study, it was found that the mechanical ventilation time, ICU stay time, and hospital stay time of the ICU-AW group were significantly higher than those of the non-ICU-AW group. This finding conformed to the conclusions of existing studies^[15,16]^ suggesting that ICU-AW patients often suffer at the same time from respiratory muscle weakness, which further prolongs weaning time, complicates the course of disease in critically ill patients, and affects their prognosis. With the extension of treatment time, the MRC-score of the ICU-AW group decreased gradually. In addition, the CT images revealed a significant reduction in the ESMcsa of the patients, suggesting the presence of significant muscle wasting. In the pathogenesis of ICU-AW, muscle protein metabolism disorder plays a vital role. Intense catabolism is an important metabolic characteristic in critically ill patients,^[17]^ especially elders, and muscle proteolysis, which is an essential part of catabolism, directly promotes the occurrence of ICU-AW.

In current clinical practice, it is generally agreed that the preferred approach for ICU-AW diagnosis is to assess the 6 major muscle groups using the MRC-score, and adopt an “aggregate score < 48” as the diagnostic criterion.^[18]^ However, the disturbance of consciousness in patients and sedative treatment in the ICU have greatly narrowed the scope of its application.^[19]^ This study revealed that ESMcsa Loss and ESMcsa Loss Ratio have certain values that can be used for the clinical diagnosis of ICU-AW. More specifically, their AUCs were 0.835 and 0.889, respectively, which suggested that they can be used as objective indicators in the clinical diagnosis of ICU-AW. In the pathogenesis of ICU-AW, acute reduction of skeletal muscle mass is an important source of muscle weakness.^[20]^ Erector spinae muscles are major antigravity skeletal muscles in the human body. Clinical studies^[9,21]^ have found that the ESMcsa can objectively reflect the skeletal muscle mass and function of patients, and is also associated with the prognosis of chronic obstructive pulmonary disease, pulmonary fibrosis, and other pulmonary diseases. As an indicator that reflects the physical activity and physiological severity of patients with pulmonary diseases, ESMcsa can serve as a highly relevant risk factor of death among chronic obstructive pulmonary disease patients.^[9,21]^ In this study, patients in the ICU who suffered from acute consumptive disease, malnutrition, and long-term bed rest, were prone to acute muscle wasting, which further led to skeletal muscle atrophy and acute degradation of muscle function and strength. In patients with mechanical ventilation, more advantages can be gained through ESMcsa measurements around the T-12 vertebra using erector spinae muscles instead of other skeletal muscles, such as pectoralis major muscles, quadriceps femoris muscles, and psoas major muscles. This is due to that the ESMcsa can be measured without taking the positions or postures of the upper and lower limbs into consideration, and can be determined during chest CT, without requiring any other scans or X-ray exposure.^[22,23]^ In addition, the ESMcsa is somewhat correlated with the MRC-score (which reflects the muscle function of patients), and various indicators offer complementary advantages. In particular, when it comes to patients in coma or under sedative treatment in the ICU, where the muscle strength cannot be assessed by the MRC-score, the ESMcsa can be employed to evaluate and predict early whether a patient is susceptible to ICU-AW.^[24]^

Moreover, it was discovered that the ESMcsa of the patients exhibited a substantial decrease within a short period, which could suggest a poorer 60-day prognosis and was probably consistent with the increased mortality of patients with combined ICU-AW in clinical practice. The results suggest that acute muscle wasting seriously deteriorates the survival quality of critically ill patients, affects their prognosis, and is attributable to the systemic multiple organ dysfunction syndrome, nutritional and metabolic imbalance, malnutrition, consumptive disease, and abruptly reduced exercise.^[25]^ Furthermore, in patients with acute respiratory failure, the respiratory muscle weakness prolongs the mechanical ventilation time, increases the probability of ventilator-associated pneumonia and other complications, and increases the failure rate of endotracheal intubation or extubation.^[26]^ Intensified weakness is associated with factors such as bed-rest time and bed-rest complications.

### 4.1 Study limitations

As an observational study, this study demonstrated only the presence of a certain correlation between acute ESMcsa reduction and acute muscle wasting, which means that it is still necessary to exclude interference factors, such as intramuscular fat content. In addition, considering that this is a single-center, small-sample study with a limited number of cases, the ESMcsa values for diagnosing ICU-AW should be further confirmed through a multicenter, large-sample clinical study.

## 5 Conclusions

The ESMcsa Loss in patients with mechanical ventilation in the ICU suggests the presence of muscle wasting and skeletal muscle hypofunction, and offers a desirable objective indicator for the diagnosis of ICU-AW. Acute ESMcsa Loss further implies a poorer prognosis, and provides certain values for predicting the 60-day survival status of patients with mechanical ventilation in the ICU.

## Author contributions

**Data curation:** Gang Yuan, Jie Zhang, Zhifang Mou.

**Formal analysis:** Gang Yuan, Jie Zhang, Jiye Luo.

**Funding acquisition:** Yongpeng Xie.

**Methodology:** Yongpeng Xie, Jie Zhang.

**Project administration:** Jiye Luo.

**Supervision:** Jiye Luo.

**Writing – original draft:** Gang Yuan, Jie Zhang, Zhifang Mou.

**Writing – review & editing:** Yongpeng Xie, Jiye Luo.
